# Knowledge, practices, and emotional experiences of Italian midwives and healthcare professionals in termination of pregnancy for fetal anomaly: The RESPeC-ToP Study

**DOI:** 10.18332/ejm/222587

**Published:** 2026-06-26

**Authors:** Claudia Ravaldi, Laura Mosconi, Anna Adami, Roberto Bonaiuti, Sara Colosi, Martina Dardari, Francesca Frati, Elena Facenda, Simona Fumagalli, Serena Neri, Antonella Nespoli, Federica Poletto, Alfredo Vannacci

**Affiliations:** 1 Department of Neurosciences, Psychology, Drug Research and Child Health, Perinatal Research Laboratory PeaRL, University of Florence, Florence, Italy; 2 CiaoLapo Foundation for Healthy Pregnancy, Stillbirth and Perinatal Loss Support, Prato, Italy; 3 School of Midwifery, Department of Medicine and Surgery, University of Verona, Verona, Italy; 4 Department of Medicine and Surgery, University of Parma, Parma, Italy; 5 School of Medicine and Surgery, University of Milano-Bicocca, Monza, Italy; 6 Department of Obstetrics, Fondazione IRCCS San Gerardo dei Tintori, Monza, Italia; 7 S. Chiara Hospital, ASUIT – Trentino Integrated University Healthcare Agency, Trentino, Italy

**Keywords:** midwives, follow-up care, termination of pregnancy for fetal anomaly, bereavement care, memory making

## Abstract

**INTRODUCTION:**

Termination of pregnancy for fetal anomaly is associated with profound psychological distress for parents and can be emotionally demanding for healthcare professionals. Evidence on professionals’ knowledge, practices, and support needs in Italy remains limited. We aimed to examine Italian healthcare professionals’ knowledge of legal aspects, bereavement care practices, and emotional experiences related to care in the context of termination of pregnancy (ToP) for fetal anomaly, and to explore factors associated with supportive behaviors.

**METHODS:**

A cross-sectional web-based survey was conducted among healthcare professionals between September 2022 and December 2023. The survey included sociodemographic and professional characteristics, knowledge of Italian law, bereavement care practices, and professionals’ emotional experiences.

**RESULTS:**

A total of 552 respondents participated of which 84.8% were midwives; 99.4% correctly identified when termination of pregnancy can be performed, while 19.5% were aware that no legally fixed gestational threshold exists; younger and less experienced respondents showed lower levels of legal knowledge (p<0.001). Feelings of inadequacy were reported by 51.4% of respondents, and 71.2% reported the need for debriefing after assisting with a termination of pregnancy. The presence of a shared emotional support protocol was significantly associated with supportive behaviors (p≤0.003). Routine use of memory boxes was reported by 53.0% of respondents, whereas follow-up care was least consistently ensured (37.4%); 97.2% recognized the need for lactation management after termination of pregnancy for fetal anomaly.

**CONCLUSIONS:**

The RESPeC-ToP Study highlights gaps in legal knowledge and uneven implementation of key bereavement care components. Training and shared emotional support protocols emerge as actionable strategies to standardize supportive practices and strengthen both quality of care and staff support within maternity services.

## INTRODUCTION

Termination of pregnancy for fetal anomaly represents a complex and emotionally demanding aspect of maternity care. In Europe, more than two thousand families each year experience a termination following the diagnosis of a fetal anomaly, with a prevalence of approximately 3.4 per 1000 births before 24 weeks of gestation^1^. In Italy, over four thousand terminations after 12 weeks of gestation were recorded in 2021^2^.

Beyond its clinical implications, termination of pregnancy for fetal anomaly has profound psychological consequences for women and their partners. Parents often experience shock, fear, guilt, and despair, compounded by the need to make rapid and consequential decisions^3-6^. A growing body of literature links this experience to prolonged grief, depressive symptoms, anxiety, and post-traumatic stress reactions^7-10^. In this context, respectful, continuous, and non-judgmental care is essential to mitigate distress and support parents during hospitalization and after discharge.

Despite this, bereavement care following termination of pregnancy remains underexplored and inconsistently implemented^11-13^. Women frequently report unmet needs in communication, emotional support, continuity of care, and follow-up^11,12,14^. Respectful care – acknowledging grief, supporting decision-making, preserving dignity, and offering meaningful choices – has been identified as central to parents’ experiences^14^. However, abortion-related stigma continues to influence care provision and care-seeking behaviors^15-17^.

Several countries have introduced structured pathways to improve care after termination of pregnancy for fetal anomaly, although the legal and organizational frameworks differ substantially. In the United Kingdom, where legislation allows flexibility regarding gestational limits in cases of severe fetal anomaly, national bereavement care guidance has promoted relatively standardized clinical pathways, including individualized support, memory-making opportunities, clear information about funeral arrangements, and access to psychological and emotional care^18,19^. In Ireland, where legislation about termination of pregnancy for fetal anomaly is more recent and more narrowly codified, national standards have similarly incorporated bereavement care principles, emphasizing multidisciplinary collaboration and shared protocols^20,21^.

In Italy, the organizational and legal context presents additional challenges. Although termination of pregnancy is regulated by Law 194/1978, no fixed gestational threshold is specified for fetal anomaly^22^. Unlike the UK context, however, this legal flexibility has not been accompanied by equally structured and widely implemented clinical pathways. As a result, clinical practice is often shaped by local interpretations related to fetal viability, maternal risk, and organizational culture, contributing to marked variability in care provision^1^. Limited knowledge of legal and administrative procedures may affect counseling, referral timing, and access to options such as burial, memory making, and follow-up care^12^.

Healthcare professionals involved in this care, particularly midwives, face significant emotional demands. Supporting parents through diagnosis, labor, birth, and the postpartum period requires high emotional engagement, while stigma may also affect professionals^23-25^. Studies report emotional burden, compassion fatigue, and burnout among those working with perinatal loss^26-28^. Training and shared clinical and emotional support protocols are key to promoting supportive practices and protecting staff wellbeing^29,30^. Structured guidance may facilitate sensitive communication, reduce paternalism, and support shared decision-making, which is associated with better parental outcomes^31^. Specific aspects of care, including memory making, follow-up planning, and lactation management, are recognized as integral but remain unevenly addressed^18,32-34^.

Within this framework, the RESPeC-ToP (Respectful Care in Termination of Pregnancy) Study was designed to explore Italian healthcare professionals’ knowledge, practices, and emotional experiences in this area of care. The study aims to: 1) assess knowledge of legal and administrative aspects of termination of pregnancy for fetal anomaly; 2) describe bereavement care practices; 3) examine emotional experiences when assisting termination of pregnancy for fetal anomaly; and 4) explore whether age, experience, training, and shared protocols are associated with supportive care behaviors.

## METHODS

### Study design and participants

The RESPeC-ToP Study adopted a cross-sectional, web-based survey design. Healthcare professionals were eligible to participate if they were involved in the care of women and couples undergoing termination of pregnancy for fetal anomaly, either in hospital settings or in community-based services (e.g. maternity clinics, outpatient services, private practice).

The survey was distributed between September 2022 and December 2023 through the online channels of the CiaoLapo Foundation, an Italian non-profit organization dedicated to research, education, and support in perinatal health and perinatal loss, in collaboration with several Italian hospitals. The Foundation’s communication channels reached approximately 67000 social media followers, a newsletter of approximately 25000 contacts, and a dedicated mailing list of approximately 7000 perinatal healthcare professionals. Eligible professionals were invited to participate and encouraged to extend the invitation to colleagues working in settings providing care for women undergoing termination of pregnancy for fetal anomaly. The survey was voluntary and anonymous; no personal data were recorded, and in no way was it possible to identify individual respondents. Informed consent was provided at the start of the survey once participants had read the participant information and met the eligibility criteria. Although no sex or gender restrictions were applied, all respondents identified as female.

### Survey development and measures

The RESPeC-ToP survey was developed by CR, AV, SN, MD, and FF, revised and pilot-tested by LM and RB, and administered using the Qualtrics online platform, provided by the Perinatal Research Laboratory (PEARL) of the University of Florence. The questionnaire was specifically designed to explore healthcare professionals’ knowledge, practices, and experiences concerning care in the context of termination of pregnancy for fetal anomaly. It consisted of four main sections.

*Sociodemographic and professional characteristics*

These included age, professional role, geographical area of practice, years of work experience, number of cases of termination of pregnancy for fetal anomaly assisted per year, and conscientious objection status.

*Knowledge of Italian legislation and administrative procedures related to termination of pregnancy for fetal anomaly*

These included awareness of legal requirements after 90 days of gestation, understanding of gestational limits, and identification of procedures from which conscientious objectors may legally abstain.

*Bereavement care knowledge and practices*

These covered topics such as burial and funeral procedures, memory making (including the use of memory boxes), follow-up organization, psychological support, and lactation management after termination of pregnancy for fetal anomaly.

*Emotional and professional experiences*

These explored healthcare professionals’ emotional responses when assisting couples (e.g. empathy, helplessness, inadequacy), perceived challenges in care provision, coping strategies, and perceived support needs. Several items allowed multiple responses. Open-ended questions were included to capture participants’ perspectives on training needs, ethical challenges, and perceived gaps in care.

### Definition of key variables

To assess adherence to legal and procedural requirements, a composite variable labeled ‘adequate assistance at admission’ was constructed. This variable was coded as 1 if the respondent correctly identified all procedures from which conscientious objectors are legally allowed to abstain, and 0 if at least one item was incorrectly identified. This indicator was used as a proxy measure of adequate legal and procedural knowledge at the time of patient admission. The presence of shared protocols was assessed by asking participants whether their workplace had written protocols or operating instructions for care relating termination of pregnancy for fetal anomaly, distinguishing between protocols addressing clinical aspects only and those covering both clinical and emotional support. Supportive care behaviors (e.g. sitting next to the woman or couple, being available to talk, staying physically close, allowing the couple to remain together, offering psychological support) were assessed through self-reported items referring to usual clinical practice.

### Statistical and qualitative analysis

Survey responses were downloaded from Qualtrics and imported into Stata/BE version 18 (StataCorp, College Station, TX, USA) for analysis. Incomplete questionnaires were excluded from the analysis. Descriptive statistics were used to summarize the data. Categorical variables were reported as frequencies and percentages and compared using the chi-squared test. Continuous variables were reported as means with standard deviations (SD) or as medians with interquartile ranges (IQR), as appropriate. Group comparisons were performed using Student’s t-test, Mann–Whitney U test, or Kruskal–Wallis test, depending on data distribution. Multivariable logistic regression was additionally performed for selected outcomes, adjusting for age, years of experience, geographical zone, conscientious objector status, and annual TOPFA (ToP for fetal anomaly) volume. Associations between training, shared protocols, and supportive behaviors were explored using bivariate analyses. Statistical significance was set at p<0.05.

Responses to open-ended questions were analyzed using a thematic approach. An initial AI-assisted coding process was applied following the methodology described by Ravaldi et al.^35^. Codes, categories, and themes were developed, reviewed, refined, and discussed by the research team through manual coding and iterative qualitative analysis. MAXQDA 2018 (VERBI Software GmbH, Berlin, Germany) was used to support data management, coding organization, and retrieval of textual data. Particular attention was paid to ethical positions and clinical attitudes related to conscientious objection and bereavement care. Graphs and figures were generated using Tableau Desktop version 2024.1 (Tableau Software, LLC).

## RESULTS

### Sample characteristics

A total of 552 healthcare professionals completed the survey. All respondents identified as female. The majority were midwives (84.8%), followed by nurses and other healthcare professionals involved in care in the context of termination of pregnancy for fetal anomaly. The mean length of professional experience was 11.2 years (SD=9.1). Overall, 20.0% of respondents identified as conscientious objectors, while 3.2% preferred not to disclose their position. Most respondents worked in Northern Italy (47.6% in the North-East and 31.9% in the North-West), followed by Central Italy (15.9%), and Southern Italy and Islands (4.5%). Nearly half of the sample (48.6%) reported assisting with five or more second-trimester cases per year. Detailed sociodemographic and professional characteristics of the sample are presented in Table 1.

**Table 1:** Sociodemographic and professional characteristics of healthcare professionals participating in the RESPeC-ToP Study, Italy, 2022– 2023 (N=552)

*Characteristics*	*n (%)*
**Sex**	
Female	552 (100)
**Age** (years)	
20–31	190 (34.4)
32–38	183 (33.2)
>38	179 (32.4)
**Profession**	
Midwife	468 (84.8)
Nurse	30 (5.4)
Medical doctor	32 (5.8)
Other*	22 (4)
**Years of work experience**	
<5	185 (33.5)
6–13	193 (35)
>13	174 (31.5)
**Conscientious objection**	
Yes	105 (20)
No	402 (76.7)
Not known	17 (3.2)
**Yearly ToP assisted**	
Never	38 (7.3)
<5	231 (44.1)
5–10	165 (31.5)
10–20	50 (9.5)
>20	40 (7.6)
**Attended courses on bereavement care**	
Yes	233 (64)

Data derived from a cross-sectional web-based survey of 552 healthcare professionals involved in hospital and community care for women and couples undergoing termination of pregnancy for fetal anomaly.

* Includes healthcare assistants and lactation consultants. ToP: termination of pregnancy.

### Knowledge of Italian legislation and administrative procedures

Almost all respondents (99.4%) correctly identified that termination of pregnancy can be legally performed after 90 days of gestation under Italian law. However, 19.5% were aware that Italian legislation does not establish a fixed gestational threshold for termination of pregnancy for fetal anomaly. The majority of respondents believed that a gestational limit exists at 22 weeks (53.7%) or 24 weeks (24.7%), while 2.1% reported not knowing the answer; 59.1% of participants correctly identified all procedures from which conscientious objectors are legally exempt (Supplementary file Table 1), thus meeting the criteria for ‘adequate assistance at admission’. The procedures most frequently (and incorrectly) identified as objectionable included childbirth assistance (28.6%), surgical care (70.2%), and pharmacological administration (95.0%). Knowledge of the legal framework differed significantly by age and professional experience. Respondents aged 20–31 years showed lower levels of legal knowledge compared with those aged 32–38 years and those >38 years (p<0.001). Similarly, healthcare professionals with <5 years of work experience were significantly less knowledgeable than those with longer professional experience (p<0.001). Respondents assisting with <5 cases of termination of pregnancy for fetal anomaly per year were more likely to report that parents should always be informed about burial options even if not explicitly requested (19.4% vs 9.1%, p<0.05).

### Knowledge and implementation of bereavement care practices

*Training and perceived importance of bereavement care*

Nearly two-thirds of respondents (64.0%) had attended at least one course on bereavement care. Interest in further training was high, with 94.7% expressing willingness to attend additional courses and 83.2% supporting the introduction of regular multidisciplinary meetings. More than half of the sample (56.9%) also expressed interest in participating in parent self-help or peer-support groups to better understand bereavement processes. Thematic analysis of open-ended responses highlighted several perceived reasons for the importance of bereavement care training: lack of formal education during professional training, the need for continuous updating, difficulties in communication, fear of making mistakes, and the emotional impact of caring for bereaved parents. Training was described as essential to improve empathy, provide accurate information, manage emotional involvement, and ensure consistent care across professionals.

*Burial procedures and memory making*

Almost all respondents (97.1%) were aware that parents can request burial services, and 90.3% believed that this request is possible under all circumstances. However, 12.1% reported that information about burial and funeral procedures is not routinely provided unless parents explicitly request it. Nearly half of respondents (49.4%) were unaware that different administrative pathways apply to burial requests when termination of pregnancy for fetal anomaly occurs before 20 weeks of gestation. Only 48.5% knew that hospitals or municipalities may independently decide to proceed with burial if parental rights are waived, while 12.0% reported uncertainty about whether such requests could be refused. Routine use of a memory box was reported by 53.0% of respondents. The memory box was perceived as highly relevant for supporting the grieving process (mean score=4.5 on a 1–5 Likert scale), with significantly higher ratings among those who had direct experience using it compared with those who had not (4.6 vs 4.4; p<0.05). Respondents also recognized the broader symbolic and anthropological value of memory making (mean score=4.2).

*Clinical practices and supportive behaviors*

Regarding the presence of shared protocols, 57.4% of respondents reported having protocols addressing clinical aspects only, while 28.9% reported protocols covering both clinical and emotional support. A minority were unsure (8.8%) or reported the absence of any protocol (5.0%). Respondents reported frequent implementation of practices aimed at preserving privacy and emotional support. Respect for the privacy of the woman or couple was reported by 91.8% of participants, and 95.9% reported offering parents the opportunity to see the baby. Emotional support during labor and birth was provided by 88.2%, and one-to-one midwifery care by 70.3%. In contrast, some practices were less consistently implemented. Labor analgesia without benzodiazepines or tranquilizers was reported by 58.5%, memory making by 62.4%, and follow-up care by only 37.4%. Notably, 38.5% reported that no follow-up was ensured, and 24.2% were unsure. The presence of a shared emotional support protocol was significantly associated with supportive behaviors. Respondents working in settings with such protocols were more likely to report sitting next to the woman or couple (93.3% vs 69.2%, p=0.003), being available to talk (98.0% vs 76.9%, p=0.002), staying physically close (96.9% vs 84.6%, p<0.001), allowing the couple to remain together (100% vs 92.3%, p<0.001), and offering psychological support (99.5% vs 92.3%, p=0.001).

In multivariable logistic regression adjusting for age, years of professional experience, geographical zone, conscientious objector status, and annual TOPFA volume, the presence of a shared emotional support protocol remained independently associated with avoiding benzodiazepines (AOR=3.96; 95% CI: 2.09–7.50), memory making (AOR=4.09; 95% CI: 2.09–7.99), postpartum support (AOR=3.60; 95% CI: 1.44–9.01), and systematic follow-up (AOR=3.83; 95% CI: 2.14–6.87). The association with one-to-one care was attenuated after adjustment and did not reach statistical significance (AOR=1.78; 95% CI: 0.97–3.26; p=0.062). Adequate assistance at admission was significantly less frequent among providers assisting with fewer than five cases per year compared with those with greater experience (4.8% vs 10.9%; χ²=14.9; p=0.005).

### Emotional experiences of healthcare professionals

Healthcare professionals reported a wide range of emotional responses when assisting with the termination of pregnancy for fetal anomaly. Empathy, emotional pain, and helplessness were the most commonly reported feelings (Table 2). More than half of respondents (51.4%) expressed feelings of inadequacy, and 67.8% described a high level of identification with parents’ experiences. Emotional involvement was more frequently reported by respondents who had attended bereavement care training (94.9% vs 86.9%; χ²=12.2; p=0.016). Empathy was also significantly more prevalent among trained professionals (71.1% vs 22.1%; χ²=18.9; p=0.001). Younger age and shorter professional experience were associated with higher emotional vulnerability. Emotional disengagement or ‘emotional anesthesia’ was reported more frequently by respondents aged 20–31 years compared with older age groups (p=0.027). Feelings of impotence and inadequacy were significantly more common among respondents with <5 years of professional experience (p=0.029 and p=0.006, respectively). After assisting a termination of pregnancy for fetal anomaly, 71.2% of respondents reported feeling the need to talk to someone, most often a colleague (88.9%), followed by family members (46.0%) and psychologists (18.0%). Communication with parents, postpartum support, and assistance during birth were identified as the most challenging aspects of care.

**Table 2:** Emotional experiences and relational responses of healthcare professionals assisting termination of pregnancy for fetal anomaly in the RESPeC-ToP Study, Italy, 2022–2023 (N=552)

*Domain*	*Item*	% (n)*
**Emotional burden and vulnerability**	Emotional distress	47.9 (220)
	Emotional pain	81.3 (373)
	Feelings of inadequacy	51.4 (236)
	Feelings of impotence	65.1 (299)
	Anger	21.1 (97)
	Emotional anesthesia	20.0 (92)
	Emotional withdrawal	12.2 (56)
	Indifference	2.0 (9)
**Emotional involvement and identification**	Emotional involvement	90.2 (414)
	Identification with parents	67.8 (311)
	Silence as emotional response	48.8 (224)
**Empathy and relational presence**	Empathy	93.9 (431)
	Sitting beside the woman/couple	91.3 (419)
	Staying in the room	83.4 (383)
	Physical proximity	96.1 (441)
**Supportive communication**	Active listening	97.2 (446)
	Showing interest and attention	97.6 (448)
	Availability to talk	95.9 (440)
	Understanding and emotional support	96.1 (441)

Data derived from a cross-sectional web-based survey of 552 healthcare professionals involved in hospital and community care for women and couples undergoing termination of pregnancy for fetal anomaly.

* Percentages refer to valid responses (‘Yes’). Missing and non-applicable responses are not shown.

### Follow-up care

The vast majority of respondents (90.4%) considered scheduled follow-up visits essential, and 97.8% supported the activation of outpatient services. Psychological support was deemed crucial by 98.4% of respondents. However, a minority considered personal phone calls (27.5%) or avoidance of discussion (9.9%) useful, and very few endorsed encouraging immediate new pregnancies (6.3%). While 49.2% supported an increased number of follow-up consultations compared with physiological postpartum care, only 26.1% considered a single gynecological visit sufficient. Less than half of respondents reported having established links between hospital services and community outpatient care.

### Lactation management after termination of pregnancy for fetal anomaly

**Figure 1:**
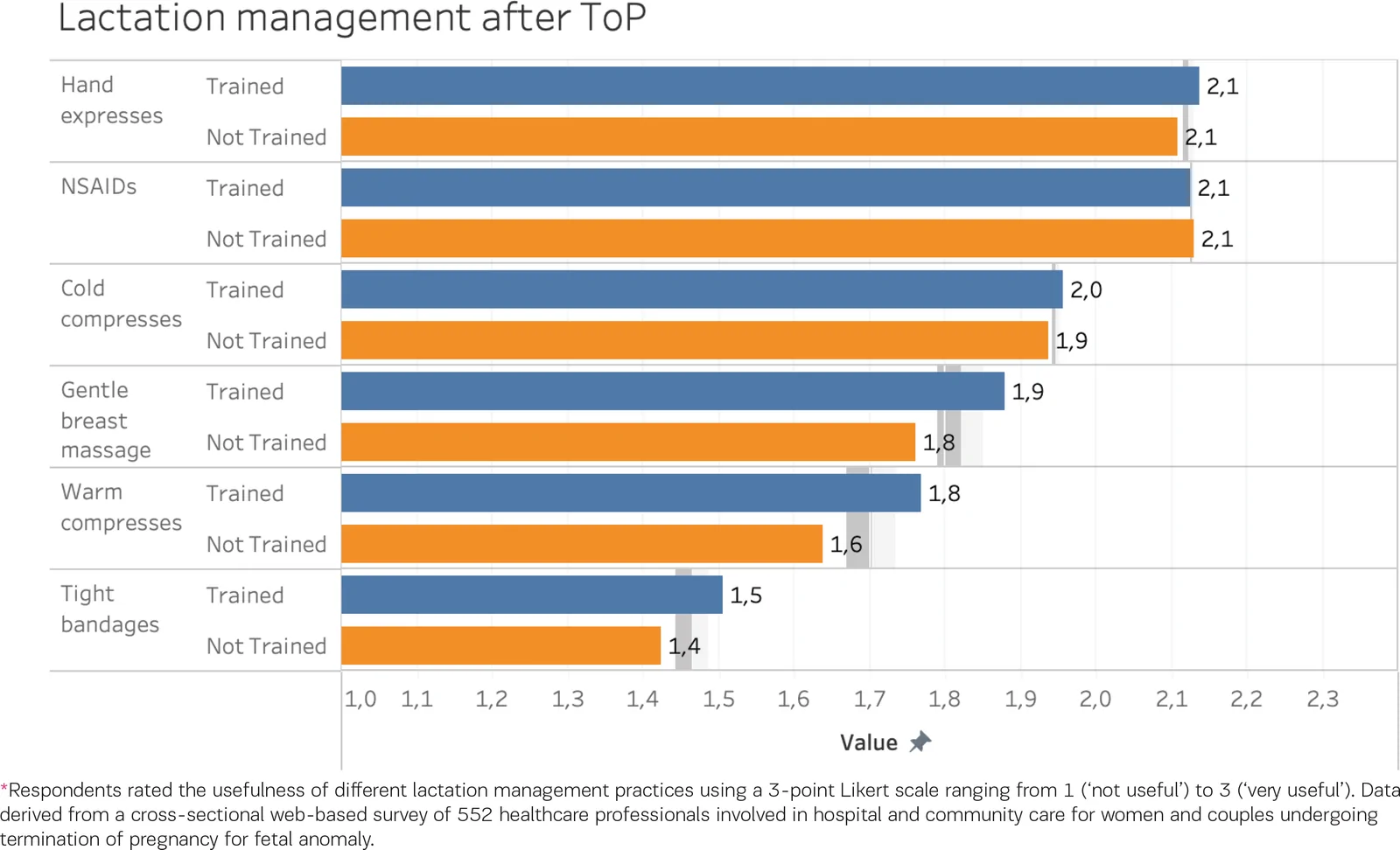
Information provided by healthcare professionals regarding lactation management* after termination of pregnancy for fetal anomaly in the RESPeC-ToP Study, Italy, 2022–2023 (N=552)

Almost all respondents (97.2%) were aware of the potential need for lactation suppression after termination of pregnancy for fetal anomaly, and 90.4% had encountered this issue in clinical practice. Although 91.5% considered providing information on lactation management important, less than half routinely discussed milk donation (33.0%) or non-pharmacological suppression methods (49.7%), while pharmacological suppression was discussed by 82.1%. Younger and less experienced professionals were more likely to report information on both pharmacological and non-pharmacological lactation management. Attendance at bereavement care training and the presence of shared emotional support protocols were significantly associated with earlier and more comprehensive provision of lactation information (Figure 1).

## DISCUSSION

The RESPeC-ToP Study provides a comprehensive overview of Italian healthcare professionals’ knowledge, practices, and emotional experiences related to the termination of pregnancy for fetal anomaly. This distribution of the sample is consistent with the concentration of Italian hospitals offering specialist obstetric services and termination of pregnancy for fetal anomaly care, and with the higher regional density of midwifery professionals, in Northern and Central Italy^36^. Our findings highlight substantial gaps in legal knowledge and uneven implementation of key bereavement care components, alongside a significant emotional burden for professionals. Importantly, training and shared clinical–emotional protocols emerged as consistent factors associated with more supportive care behaviors, identifying actionable levers to improve both quality of care and staff support.

### Legal knowledge and organizational variability

Although most respondents knew that termination of pregnancy for fetal anomaly can be performed after 90 days of gestation, only a minority correctly identified that Italian law does not establish a fixed gestational limit. This gap between legislation and perceived clinical practice is highly relevant, as misunderstandings may contribute to delayed referrals, inconsistent counseling, and unequal access to care, increasing parents’ distress during decision making^1,12^.

Younger and less experienced professionals showed lower levels of legal knowledge, suggesting that initial training may be insufficiently addressing legal and administrative aspects of care. Targeted education and clearer institutional guidance are therefore needed to reduce uncertainty and promote consistent practice. From a midwifery perspective, legal clarity directly influences the ability to provide timely, confident, and respectful care.

### Bereavement care practices: from recommendations to reality

Although international guidelines recommend comprehensive bereavement pathways after termination of pregnancy for fetal anomaly^18,20^, our findings suggest inconsistent implementation in Italian clinical practice. Privacy, emotional support during labor and birth, and opportunities to see the baby were commonly provided, whereas other key components – such as memory boxes, structured follow-up, and comprehensive lactation counseling – were less consistently offered. Memory making, despite being recognized as meaningful for grief processing, was routinely proposed by only about half of respondents, suggesting organizational or cultural barriers rather than a lack of awareness. Similar gaps emerged for follow-up care, considered essential by most participants but rarely ensured in practice. Limited continuity after discharge may increase the risk of complicated grief^11,12,14^ and leave parents feeling unsupported during a vulnerable period.

### The role of protocols and training as modifiable levers

One of the strongest findings of the study was the association between shared emotional support protocols and supportive care behaviors. Professionals working in settings with such protocols were more likely to adopt relational practices characterized by proximity, availability, and respectful midwifery care, suggesting the possibility that supportive behaviors are linked not only to individual sensitivity but also to organizational structures^29,30^. Bereavement care training also emerged as a key factor: trained professionals reported greater empathy, emotional engagement, and more comprehensive management of sensitive issues such as lactation. Rather than reflecting increased vulnerability, this may indicate greater emotional awareness and professional attunement, which are central to midwifery care. Structured training may therefore improve both the quality of care and professionals’ ability to recognize and manage their emotional responses^30,31^.

### Emotional burden and professional vulnerability

Consistent with previous literature^27,28^, healthcare professionals reported high emotional burden when assisting with the termination of pregnancy for fetal anomaly. Feelings of empathy, pain, helplessness, and inadequacy were particularly common among younger and less experienced staff, highlighting the emotional impact of care in contexts of loss, moral complexity, and stigma. More than half of respondents reported feeling inadequate, and most expressed the need for debriefing, mainly with colleagues, indicating that peer support represents an important but largely informal coping strategy. Without adequate organizational support, repeated exposure to these situations may increase the risk of compassion fatigue, secondary traumatic stress, and burnout^26-28^. Integrating structured reflection, supervision, and peer support into routine midwifery practice may therefore be essential to sustain compassionate care over time.

### Lactation management as an overlooked component of bereavement care

Lactation management after termination of pregnancy for fetal anomaly emerged as a sensitive and often under-addressed aspect of care. Although most professionals recognized its importance, information provided to women mainly focused on pharmacological suppression, with less attention to non-pharmacological options or milk donation. This may reflect discomfort, limited time, or insufficient training rather than a lack of clinical relevance. Younger and specifically trained professionals were more likely to provide comprehensive information, suggesting that education may reduce avoidance and emotional distancing. Open and sensitive management of lactation may help reduce physical discomfort while also supporting women’s emotional processing of loss, reinforcing the holistic dimension of midwifery care^33^.

### Implications for midwifery practice and future research

Our findings suggest that care following termination of pregnancy for fetal anomaly depends not only on individual resilience, but also on the presence of structured organizational support. Clear protocols, targeted training, and integrated follow-up pathways appear associated with lower professional uncertainty and greater emotional sustainability. Future research should examine the implementation and effectiveness of structured bereavement pathways through observational and longitudinal studies, and further explore the links between emotional burden, secondary traumatization, and quality of care. Involving midwives in the co-design of protocols and training programs may help bridge the gap between recommendations and clinical practice.

### Strengths and limitations

The RESPeC-ToP Study presents several strengths. It is among the largest studies exploring healthcare professionals’ knowledge, practices, and emotional experiences regarding termination of pregnancy for fetal anomaly in Western countries. The large sample, mainly composed of midwives, offers a practice-oriented overview of routine care. The study also adopts an integrated approach, examining legal knowledge, clinical and organizational practices, emotional experiences, and training factors, thus reflecting the complexity of care in this context. The inclusion of often-neglected aspects such as follow-up, memory making, and lactation management enhances its clinical relevance and provides novel contributions to the literature.

Some limitations should be considered. The cross-sectional design precludes causal inferences. Data were collected through a voluntary web-based survey, potentially introducing selection and self-report bias, and over-representing professionals interested in bereavement care practices and experiences, which were not directly observed. The geographical distribution of the sample reflects the concentration of Italian perinatal services and the midwifery workforce in Northern and Central Italy; participation from Southern Italy and the Islands was more limited, which may restrict the generalizability of findings to those regions. As no sampling frame exists for Italian professionals involved in the termination of pregnancy for fetal anomaly, response rates could not be calculated. The questionnaire, although based on literature and guidelines, was developed by the research team and not psychometrically validated. The use of AI-assisted coding for qualitative data analysis, while appropriate for managing the large volume of responses, may have prioritized consistency and breadth over the interpretive depth achievable through fully manual qualitative approaches; qualitative findings should therefore be interpreted within these boundaries. The binary coding of the ‘adequate assistance at admission’ variable may not fully capture procedural knowledge. In addition, no correction for multiple comparisons was applied, increasing the possibility of false-positive findings in secondary analyses. Furthermore, the postpartum support model yielded an events-per-variable ratio below the recommended threshold, potentially reducing the stability and precision of some adjusted estimates. However, a sensitivity analysis restricted to midwives (n=468) yielded consistent results, with odds ratios for the five primary protocol–behavior associations ranging from 3.79 to 6.57, supporting the robustness of the main findings.

## CONCLUSIONS

The RESPeC-ToP Study is the first investigation to explore Italian healthcare professionals’ knowledge, practices, and emotional experiences related to the termination of pregnancy for fetal anomaly. Our findings reveal important gaps in legal knowledge and uneven implementation of key components of bereavement care, including follow-up, memory making, burial procedures, and comprehensive lactation management. At the same time, the study highlights clear and actionable opportunities for improvement. Training in bereavement care and the availability of shared clinical and emotional support protocols were consistently associated with more supportive behaviors and greater professional attunement. These organizational factors appear particularly relevant for younger and less experienced professionals, who reported higher levels of uncertainty and emotional vulnerability. From a midwifery perspective, improving care in the context of termination of pregnancy for fetal anomaly requires moving beyond reliance on individual sensitivity toward structured, team-based approaches supporting both parental care and professional wellbeing. Investing in targeted education, clear protocols, and continuity of care pathways may contribute to more consistent, respectful, and sustainable care for families facing termination of pregnancy for fetal anomaly, while also protecting the wellbeing of healthcare professionals involved.

## Supplementary Material



## Data Availability

The data supporting this research are available from the authors on reasonable request.
