# Supplementary material for: Knowledge, practices, and emotional experiences of Italian midwives and healthcare professionals in termination of pregnancy for fetal anomaly: The RESPeC-ToP Study
Source: Eur J Midwifery. 2026 Jun 26;10:10.18332/ejm/222587. doi: 10.18332/ejm/222587 (PMC13573999; doi:10.18332/ejm/222587)
Supplement: Supplementary file 1 [file EJM-10-22-s1.pdf]

Supplementary Table 1. Knowledge of Italian legislation regarding procedures from which conscientious objectors are legally exempt and awareness of burial procedures after termination of pregnancy for fetal anomaly among healthcare professionals participating in the RESPeC-ToP study, Italy, 2022–2023. Data derived from a cross-sectional web-based survey of 552 healthcare professionals involved in hospital and community care for women and couples undergoing termination of pregnancy for fetal anomaly. Missing and non-applicable responses are not shown.

Variable	N (%)
**Knows all procedures that can be refuted**	
Yes	326 (59.1%)
No	226 (40.9%)
**Childbirth care**	
Yes	150 (28.6%)
No	362 (69.1%)
N.A.	12 (2.3%)
**Hospitalization bureaucracy**	
Yes	24 (4.6%)
No	490 (93.5%)
N.A.	10 (1.9%)
**Painkillers administration**	
Yes	11 (2.1%)
No	515 (98.3%)
N.A.	4 (0.8%)
**Emergency care**	
Yes	5 (1%)
No	515 (98.3%)
N.A.	4 (0.8%)
**Filling of medical records**	
Yes	21 (4%)
No	493 (94.1%)
N.A.	10 (1.9%)
**Surgery care**	
Yes	368 (70.2%)
No	142 (27.1%)
N.A.	14 (2.7%)

**RU486 administration**	
Yes	498 (95%)
No	22 (4.2%)
N.A.	4 (0.8%)
**Preoperative care**	
Yes	72 (13.7%)
No	431 (82.3%
N.A.	21 (4%)
**Knowledge about the possibility of burial**	
Yes	509 (97.1%)
**Burial is possible in any case**	
Yes	402 (90.3%)
**Different procedures under 20 WG**	
Yes	225 (50.6%)
**Giving burial information also if not requested**	
Yes	391 (87.9%)
**Hospital can bury anyway**	
Yes	216 (48.5%)
**Hospital can decide NOT to bury**	
Yes	8 (1.8%)

STROBE Statement—Checklist of items that should be included in reports of cross-sectional studies

	Item No	Recommendation	Page No
**Title and abstract**	1	(*a*) Indicate the study's design with a commonly used term in the title or the abstract	1
		(*b*) Provide in the abstract an informative and balanced summary of what was done and what was found	1
**Introduction**			
Background/rationale	2	Explain the scientific background and rationale for the investigation being reported	2
Objectives	3	State specific objectives, including any prespecified hypotheses	3
**Methods**			
Study design	4	Present key elements of study design early in the paper	4
Setting	5	Describe the setting, locations, and relevant dates, including periods of recruitment, exposure, follow-up, and data collection	4
Participants	6	(*a*) Give the eligibility criteria, and the sources and methods of selection of participants	4
Variables	7	Clearly define all outcomes, exposures, predictors, potential confounders, and effect modifiers. Give diagnostic criteria, if applicable	4-5
Data sources/ measurement	8	For each variable of interest, give sources of data and details of methods of assessment (measurement). Describe comparability of assessment methods if there is more than one group	*4-5*
Bias	9	Describe any efforts to address potential sources of bias	11
Study size	10	Explain how the study size was arrived at	4

Quantitative variables	11	Explain how quantitative variables were handled in the analyses. If applicable, describe which groupings were chosen and why	5
Statistical methods	12	(*a*) Describe all statistical methods, including those used to control for confounding	5
		(*b*) Describe any methods used to examine subgroups and interactions	5
		(*c*) Explain how missing data were addressed	5
		(*d*) If applicable, describe analytical methods taking account of sampling strategy	
		(*e*) Describe any sensitivity analyses	5
**Results**			
Participants	13	(a) Report numbers of individuals at each stage of study—eg numbers potentially eligible, examined for eligibility, confirmed eligible, included in the study, completing follow-up, and analysed	6
		(b) Give reasons for non-participation at each stage	N/A - Participation was voluntary and self-selected; no stepwise exclusions occurred beyond automated exclusion of incomplete questionnaires.
		(c) Consider use of a flow diagram	N/A - The study design did not include multiple recruitment or exclusion stages requiring a flow diagram.
Descriptive data	14	(a) Give characteristics of study participants (eg demographic, clinical, social) and information on exposures and potential confounders	6, Table 1
		(b) Indicate number of participants with missing data for each variable of interest	N/A - Records with missing data were excluded at the data extraction stage. Analyses were therefore conducted on a complete-case dataset, and the

			final analytical sample contained no missing values for the variables of interest.
Outcome data	15	Report numbers of outcome events or summary measures	6-7-8
Main results	16	(*a*) Give unadjusted estimates and, if applicable, confounder-adjusted estimates and their precision (eg, 95% confidence interval). Make clear which confounders were adjusted for and why they were included	6-7-8
		(*b*) Report category boundaries when continuous variables were categorized	6-7-8
		(*c*) If relevant, consider translating estimates of relative risk into absolute risk for a meaningful time period	N/A - No relative risk estimates were produced.
Other analyses	17	Report other analyses done—eg analyses of subgroups and interactions, and sensitivity analyses	6-7-8
**Discussion**			
Key results	18	Summarise key results with reference to study objectives	9
Limitations	19	Discuss limitations of the study, taking into account sources of potential bias or imprecision. Discuss both direction and magnitude of any potential bias	11
Interpretation	20	Give a cautious overall interpretation of results considering objectives, limitations, multiplicity of analyses, results from similar studies, and other relevant evidence	9-10-11
Generalisability	21	Discuss the generalisability (external validity) of the study results	11
**Other information**			
Funding	22	Give the source of funding and the role of the funders for the present study and, if applicable,	N/A - No external funding was received for this study. Infrastructure and logistical

	for the original study on which the present article is based	support were provided without financial contribution.

© 2026 Ravaldi C. et al.
